# Comprehensive league table of cost-utility ratios: A systematic review of cost-effectiveness evidence for health policy decisions in India

**DOI:** 10.3389/fpubh.2022.831254

**Published:** 2022-10-13

**Authors:** Komal Shah, Malkeet Singh, Priya Kotwani, Kirti Tyagi, Apurvakumar Pandya, Somen Saha, Deepak Saxena, Kavitha Rajshekar

**Affiliations:** ^1^Indian Institute of Public Health Gandhinagar (IIPHG), Gandhinagar, India; ^2^HTAIn Secretariat-Department of Health Research, New Delhi, India; ^3^Jhpiego, New Delhi, India; ^4^Faculty of Medicine, Parul Institute of Public Health, Parul University, Vadodara, India

**Keywords:** country-specific “league table”, Health Technology Assessment, India, cost-effectiveness, policy decision-making process

## Abstract

**Background and objectives:**

Although a relatively recent concept for developing countries, the developed world has been using League Tables as a policy guiding tool for a comprehensive assessment of health expenditures; country-specific “League tables” can be a very useful tool for national healthcare planning and budgeting. Presented herewith is a comprehensive league table of cost per Quality Adjusted Life Years (QALY) or Disability Adjusted Life Years (DALY) ratios derived from Health Technology Assessment (HTA) or economic evaluation studies reported from India through a systematic review.

**Methods:**

Economic evaluations and HTAs published from January 2003 to October 2019 were searched from various databases. We only included the studies reporting common outcomes (QALY/DALY) and methodology to increase the generalizability of league table findings. To opt for a uniform criterion, a reference case approach developed by Health Technology Assessment in India (HTAIn) was used for the reporting of the incremental cost-effectiveness ratio. However, as, most of the articles expressed the outcome as DALY, both (QALY and DALY) were used as outcome indicators for this review.

**Results:**

After the initial screening of 9,823 articles, 79 articles meeting the inclusion criteria were selected for the League table preparation. The spectrum of intervention was dominated by innovations for infectious diseases (33%), closely followed by maternal and child health (29%), and non-communicable diseases (20%). The remaining 18% of the interventions were on other groups of health issues, such as injuries, snake bites, and epilepsy. Most of the interventions (70%) reported DALY as an outcome indicator, and the rest (30%) reported QALY. Outcome and cost were discounted at the rate of 3 by 73% of the studies, at 5 by 4% of the studies, whereas 23% of the studies did not discount it. Budget impact and sensitivity analysis were reported by 18 and 73% of the studies, respectively.

**Interpretation and conclusions:**

The present review offers a reasonably coherent league table that reflects ICER values of a range of health conditions in India. It presents an update for decision-makers for making decisions about resource allocation.

## Introduction

The Department of Health Research of India has introduced Health Technology Assessment (HTA) processes for better allocation of the resources within the health sector and a governing body, such as HTA in India (HTAIn), is established at the Department of Health Research (DHR). Its mandate is to undertake a critical appraisal of the available technologies and identify the most cost-effective interventions. This initiative recognizes the important role of economic evidence in setting health-sector priorities (1). HTAIn aims to encourage investment in cost-effective interventions that will reduce the cost of patient care, expenditure on medical equipment, the overall cost of medical treatment, out-of-pocket expenditure, and streamline medical reimbursement procedures (2).

The contribution of economic evaluation in guiding decisions on resource allocation in health care has been widely accepted (3). In this context, the “cost per quality-adjusted life-year (QALY) gained” league table, is a well-known tool for both health economists and health care decision-makers alike (2, 3).

League-table is a great tool for stakeholders, such as policymakers, decision-makers at state and central government levels, insurance companies, and pharmaceutical companies who are working on the cost-effectiveness of their new products (drug, vaccine, or medical devices) to determine threshold values to help them assess and interpret the cost-effectiveness of the health technology under study. League tables rank health technology interventions or products or programs in terms of cost-effectiveness for numerous diseases (4, 5).

League tables rank healthcare interventions based on their incremental cost-effectiveness ratios (ICERs) and inform decision-makers about the prioritization of effective healthcare programs and allocation of scarce healthcare resources.

League tables are valuable tools for prioritizing health expenses, especially for national health resources, and have been used as a policy tool by high-, midde-, and low-income countries (6–8). League tables have been used in several prioritization exercises, such as World Bank Health Sector Priorities Review; (9, 10) the WHO Choosing Interventions that are Cost-Effective (WHO-CHOICE) initiative, (11, 12) and the World Health Report since 2000 by the WHO (13).

A few regional league tables are available for some diseases. For example, there are league tables in Africa for 60 different interventions (6). The league tables are available in other countries as well (14, 15). They have been used as policy tools for high-income, as well as low- and middle-income countries (5). Country-specific “league tables” are often a useful tool for national healthcare planning and budgeting (6, 7).

Gerald and Morrey argued that the important goal of QALY league tables is the maximization of the utility of health gains within a health service budget (16). They have stressed that league tables can be a potential means to transfer the results of the original studies to the local context. Though cost-effectiveness is not the only important criterion for policy choice, it provides a useful and comprehensible reference point.

Considering India's meager public investment in the health sector, it is critical that resources are used astutely on cost-effective interventions. Evidence-based policy decisions require robust technical evaluations and, hence, we aim to construct an all-inclusive document of cost-utility ratio findings based on peer-reviewed published research from India, obtained from standard databases. Considering the country's transition to Universal Health Coverage (UHC), priorities are set for achieving the Sustainable Development Goals (SDGs), and with the inception of HTAIn, it is timely to assess that the evidence published around the cost-effectiveness of healthcare interventions, including programs in India to avoid duplication of efforts, identify and prioritize HTA study areas, and, importantly, develop a league table for the country.

In 2015, Prinja et al. (17) conducted a systematic review of economic evaluations of healthcare interventions or program published during the period from January 1980 to mid of November 2014. Findings indicated the need for better economic evaluations with robust methodologies in India, as only one-third of the studies assessed modeling structural uncertainties (33%), or run sub-group analyses to account for heterogeneity (36.5%), or analyzed methodological uncertainty (32%). The aim of the study was to develop a comprehensive league table of cost per QALY or DALY ratios derived from HTA or economic evaluation studies conducted in India from January 2003 to October 2019.

## Methods

### Search strategy

Cost-effectiveness studies and health technology assessments conducted in India between January 2003 to October 2019 were searched from PubMed, Scopus, and York Center for Reviews and Dissemination. Keywords were checked for controlled vocabulary under Medical Subject Headings (MeSH) of PubMed. We selected articles published from 2003, as latter part of 2002; a revised National Health Policy in India was introduced that aimed to provide more equitable health care services across all the classes of population across country. This policy essentially focused on achieving acceptable standards of good quality health care for Indian population and, hence, a general interest to look for evidence-supported cost effective solutions started around that period. The Preferred Reporting Items for Systematic Reviews and Meta-Analysis (PRISMA) was used for this review, where “Cochrane Handbook for Systematic Reviews of Interventions version 6.1 2020,” particularly parts 2 and 3 of the handbook, was followed for conducting the review (18).

We only included studies reporting common outcomes (QALY or DALY) and methodology to increase the generalizability of league table findings. To opt for a uniform criterion, a reference case approach developed by Health Technology Assessment in India (HTAIn) was used (19). The Indian reference case encompasses guidance for conducting and reporting economic evaluation drawn from global best practices and a principle-based approach to strike a balance between specificity and flexibility for the Indian context. Although the reference case advocates reporting of incremental cost-effectiveness ratio with respect to QALY only, a large number of studies assessed cost-effectiveness in the form of DALY, and, hence, to provide a holistic overview of the Indian HTA studies, we included both QALY and DALY for the review. The review included only peer-reviewed articles that were reported in the English language. Abstracts, non-peer-reviewed reports, expert opinion, editorial and narrative reviews, partial economic evaluations, and cost analysis were excluded from the review.

### Selection of studies

Three researchers extracted records from various databases. At the initial stage, all duplicates were removed, and relevant studies were selected for evaluation by title and abstract assessment that was followed by full-text review, which involved examination of the content for key indicators of review. In the second-stage screening, only full health economic evaluations that were comparing both costs and outcomes of two or more health-care interventions or program pertaining to India, published in English during January 2003 to October 2019, were critically reviewed. At this stage, a bibliographic search of the selected studies was carried out to identify additional relevant economic evaluations. The search continued until no new article was found. A disagreement between the two authors, having access to abstracts and full text of the paper and decided on its inclusion and discrepancies between the two investigators, was resolved in discussion with the third author. Efforts were ensured to eliminate any bias by adhering to strict criteria for inclusion of studies in the review.

### Selection criteria for standardized league table and quality assessment

The cost-effectiveness reported in Indian studies varied widely in terms of quality and minimum requirements as mentioned in the reference cases recently, as developed and published by DHR. Hence, the selection criteria were restricted to few vital criteria only, adapted from previously reported guidelines (17, 19). This included information on perspective, outcome reporting (QALY/DALY), appropriate comparisons of incremental comparisons, time horizon, type of data and type of model used, and type of comparator used.

To avoid any possible criticism on heterogeneity associated with methodologies of various economic evaluations that results in the poorer utilization of league tables for policy decisions, current analysis included studies, which were critically evaluated for key indicators of quality, using the CHEERs checklist (20). Broadly, the information on sensitivity analysis, budget impact analysis, and discounting of the cost and effect were chosen as quality indicators. It is important to note that limited studies have included all these indicators. The systematic search yielded a large number of Indian studies reporting cost-effectiveness in terms of life years saved; however, to maintain uniformity and to follow reference cases, these studies were excluded from the review.

### Adjustment of published cost-utility ratios

To present generalizable findings that may hold value in policy decisions at the present scenario, the reported ICERs were indexed to 2020 US$ and were mentioned accordingly. In the case of studies reporting ICERs in terms of INR (Indian currency), first, the published INR were converted to US$ (for the published year) and then inflated to 2020 US$.

### ICER and ICER threshold assessment

The incremental cost-effectiveness ratio (ICER) is a measure to summarize the cost-effectiveness of a health care intervention in comparison with an alternative intervention or no alternative intervention. It is defined as a ratio of the difference in cost between two possible interventions, divided by the difference in their effect based on health outcomes (usually, quality adjusted life years or disability adjusted life years) (11, 21). It represents the average incremental cost associated with 1 additional unit of the measure of effect.

The cost per QALY/DALY assessed in the present study includes various modalities of threshold assessment, such as the following: (1) widely recommended approach of economic evaluation—per capita gross domestic product (GDP) was used to identify cost-effective studies for Indian health care set up; (21) and (2) Distribution of all the cost per QALY/DALY were plotted and categorized according to quartiles.

Decision-makers can use it as a decision rule in resource allocation based on a cost-effectiveness threshold. The concept of a cost-effectiveness threshold represents the highest value that society is willing to pay for a unit of health gain or forgo by funding the intervention (opportunity cost). There are many thresholds used for decision-making in a cost-effectiveness analysis, such as supply-side threshold (a measure of allocating resources from the provider's perspective), demand-side threshold (a measure of allocating resources from patient's perspective), or GDP based thresholds (willingness-to-pay value by individuals) (21). GDP-based threshold is recommended by several guidelines in the absence of evidence on other threshold measures (2, 19). Interventions below the threshold value are judged as cost-effective and usually accepted and funded, while those above the threshold value are considered too expensive.

## Results

**Figure 1:**
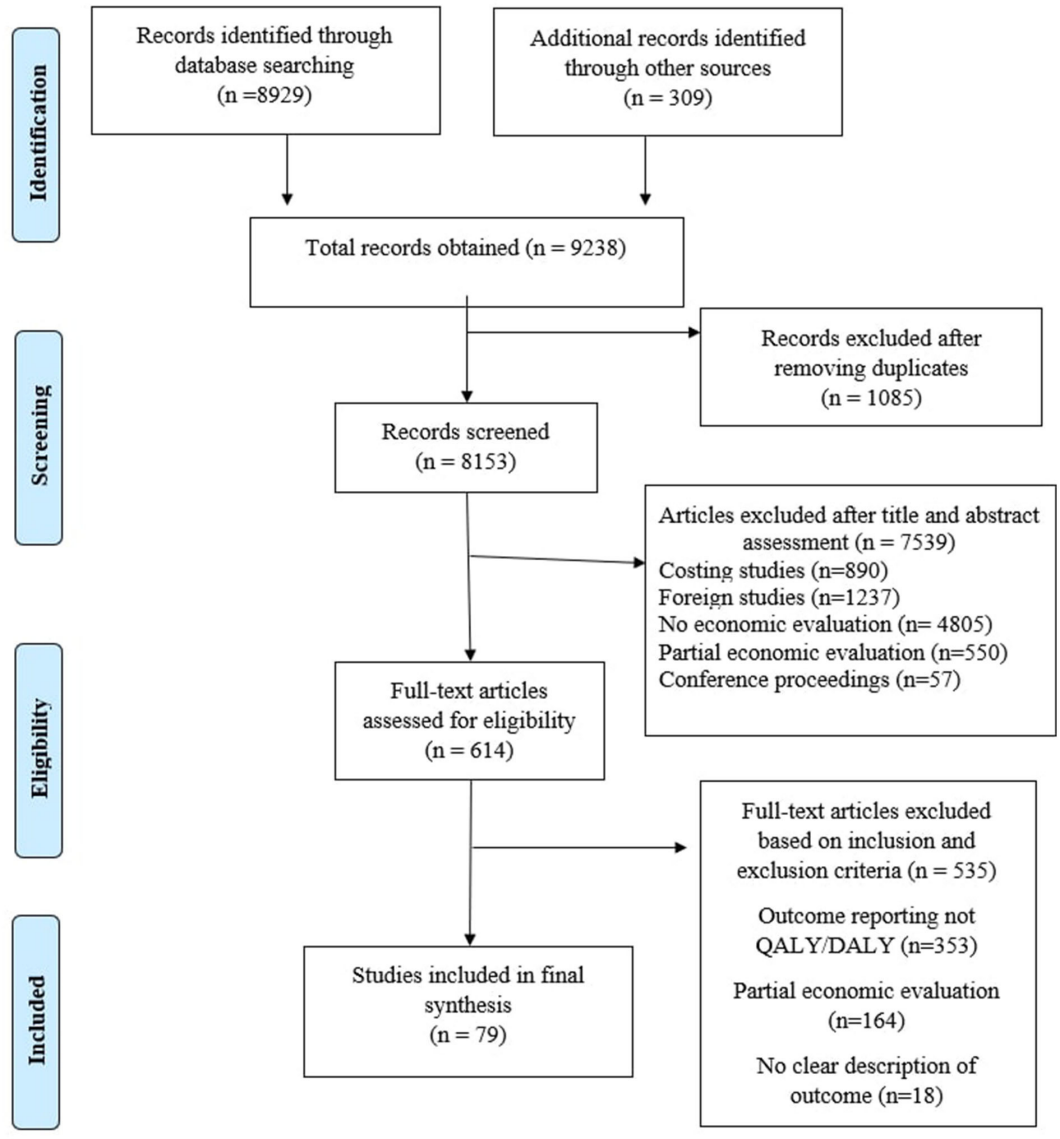
PRISMA chart.

After initial search of the previously mentioned databases, 9,238 articles were assessed for their potential inclusion in the study. The detailed strategy opted for systematic retrieval of the articles is presented in Figure 1. One thousand eighty-five articles were found as duplicated reports and were removed from the record. The remaining 8,153 articles were evaluated based on title and abstract. After careful assessment, 6,789 articles were eliminated. Full text of the shortlisted articles was retrieved and finally included in the study. Based on various reasons mentioned in the PRISMA diagram, 79 articles (13–91) were included for final evidence synthesis and league table preparation.

### Characteristics of the included studies

The key characteristics of the included studies are enlisted in Supplementary Table 1. The critical information needed to provide a league table, such as author, title of the study, publication year, type of disease, type of innovation and ICER (indexed for 2020), the status of budget impact analysis, and sensitivity analysis, were extracted from each study and is presented in a tabular format. General summary of other important features of the studies were analyzed and shown in Table 1. Studies assessing cost-effectiveness from the payer's or provider's perspective (56.25%) were dominated over by other studies considering societal perspective (43.75%), including consideration of out-of-pocket expenditure.

**Table 1:** Characteristics of the published studies reporting cost-utility ratios.

**Variables**	***N***	**Percentage**
**Study perspective**		
Payer's or Provider's perspective	45	56.96
Societal perspective	34	43.04
**Diseases**		
Infectious diseases	26	32.9
Maternal and Child health	24	29.1
Non-communicable diseases	16	20.3
Others	14	17.7
**Study comparator**		
Conventional/Routine care	50	63.3
No intervention	29	36.7
**Nature of care (Health technology)**		
Preventive	47	59.5
Curative	32	40.5
**Setting of intervention**		
Community based/Primary health care	24	30.4
Facility-based intervention	55	62.6
**Type of cost data used**		
Primary	21	26.25
Secondary	58	73.75
**Type of effectiveness data used**		
Primary	13	16.5
Secondary	66	83.5
**Cost-effectiveness of the intervention**		
Yes	79	100
**Type of model**		
Decision analytical model (Markov/Decision tree/any other)	61	77.2
Not mentioned	18	22.8
**Time horizon**		
≤1 year	8	10
2–5 years	6	7.6
>10 years but not life time	21	26.6
Life time	36	45.6
Not mentioned	8	10
**Outcome measure**		
DALY	55	69.6
QALY	24	30.4
**Discounting status**		
3%	58	73.4
5%	3	3.8
Not done	18	22.8
**Budget impact analysis**		
Yes	14	17.7
No	65	82.3
**Sensitivity analysis**		
OWSA with/without other methods	32	40.5
PSA	15	19.0
Others	7	8.9
Not mentioned type	4	5.1

Studies were broadly classified into following categories: Infectious diseases, non-communicable diseases, maternal and child health, and others. Details of the disease type distribution are also provided in the Table 1, indicating huge share of studies addressing interventions for Infectious diseases. Popular choice of comparator was routine/conventional scenario (63.3%) over absence of any intervention (36.7%). Large number of HTA studies were for preventive intervention (59.5%), followed by the curative intervention (40.5%). Both cost and clinical efficacy studies chiefly used secondary available literature and data. Almost half (46.25%) of the studies assessed the impact of intervention over a lifetime. Outcome reporting opted for the studies was predominantly disability-adjusted life years averted (70%). The majority of the studies were of good quality as indicated by CHEER's checklist-based assessment. A considerable number of the studies undertook sensitivity analysis (73.4%). However, budget impact analysis was not explored by 18% of the studies.

### ICER threshold

**Figure 2:**
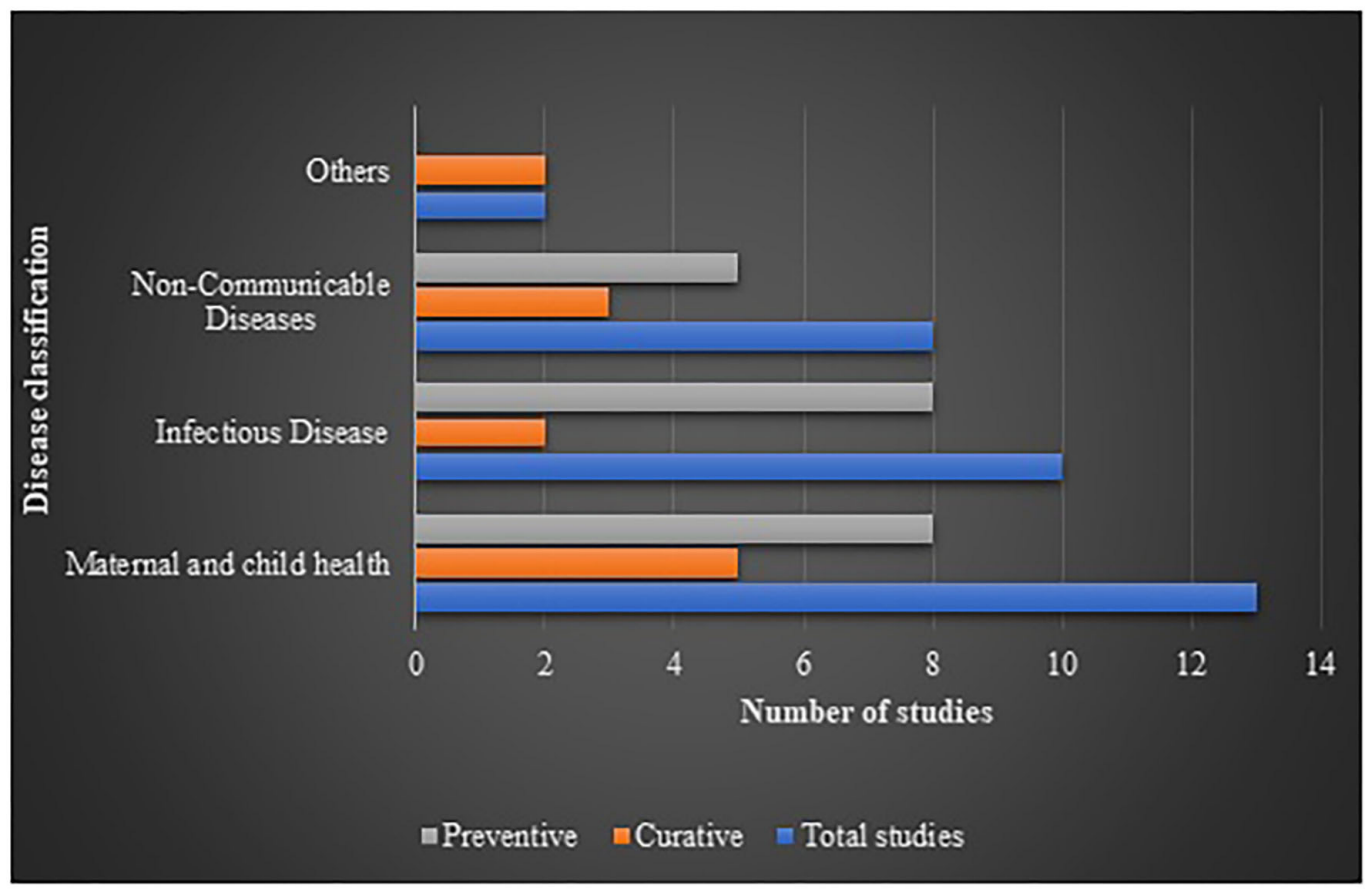
Number of HTA studies classified as per disease and nature of cure.

**Figure 3:**
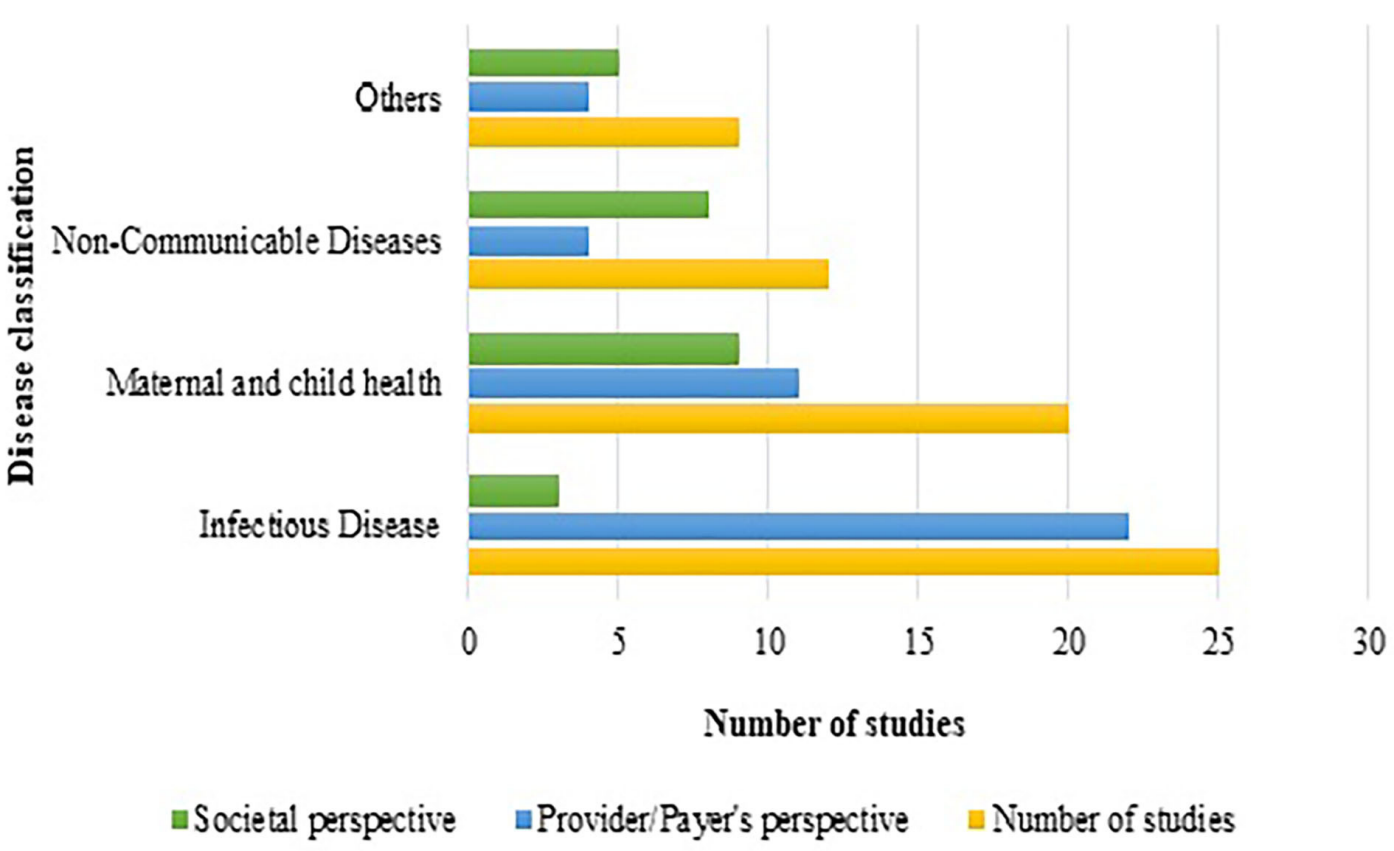
Number of HTA studies classified as per disease and perspective.

Interventions showing ICER were plotted against diseases (the diseases were broadly classified into 4 categories of interest, nature of care and shown in Figure 2. It indicated that most of the interventions addressed infectious diseases and maternal and child health, where preventive interventions preponderated the spectrum. A comprehensive assessment of ICER corresponding to per capita gross domestic product (GDP) of India (as per 2019 statistics) according to diseases and study perspective is presented as Figure 3. Sixty-six (83.5%) interventions fall below the threshold of per capita Indian GDP, where majority of the studies assessed cost-effectiveness of the innovations for maternal and child diseases and infectious diseases. Chiefly, the studies evaluated the effectiveness from provider's (healthcare services, health practitioners) and payer's perspectives (such as government, insurance, or healthcare services by the non-governmental organizations) and, hence, have not considered out-of-pocket expenditures.

**Figure 4:**
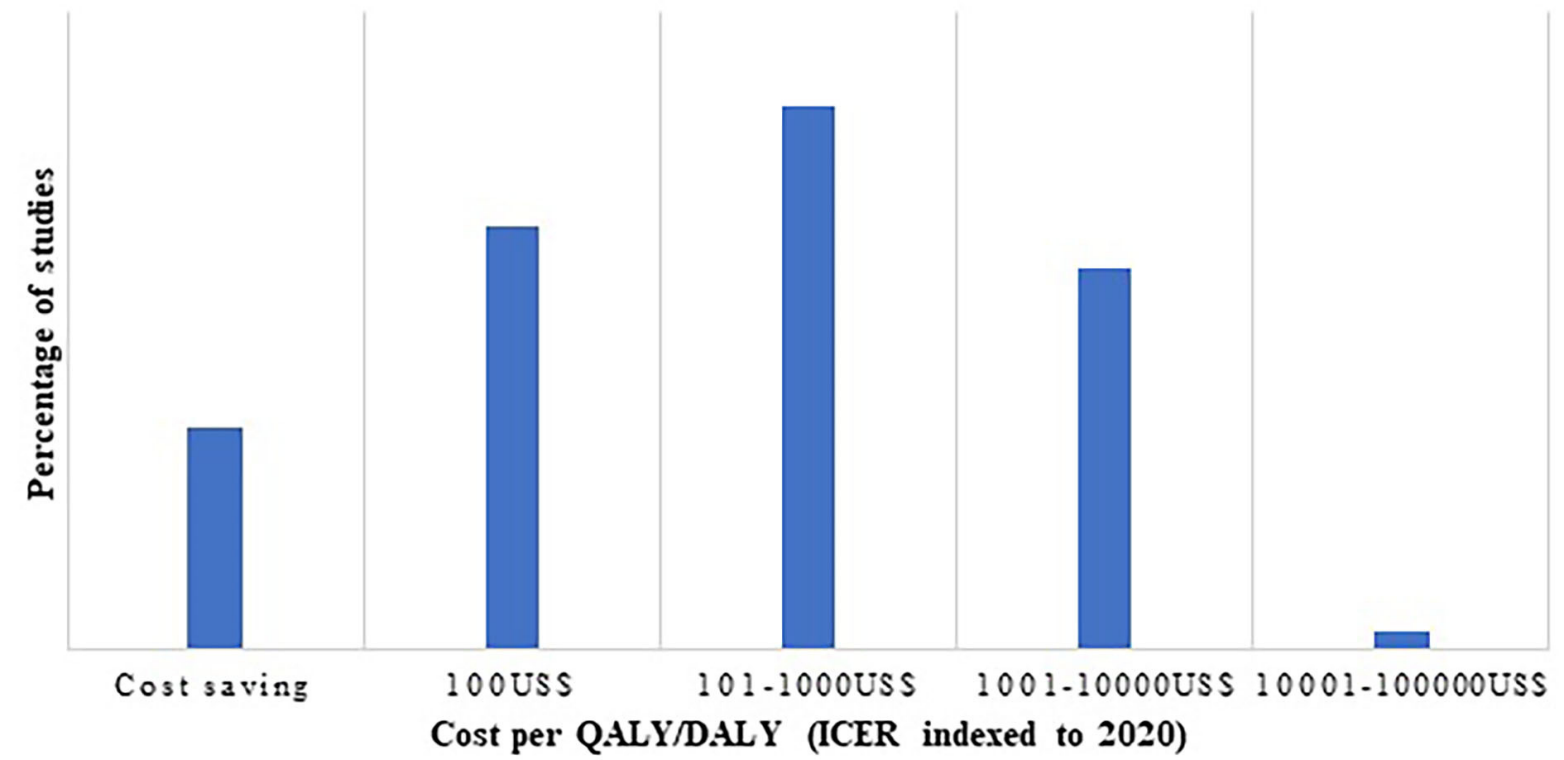
Number of studies classified as cost per QALY/DALY estimates.

As indicated in Figure 4, cost per QALY/DALY estimates were chiefly falling in the range of cost saving to 1,000 US$ per QALY/DALY. Proportion of cost saving interventions was 13.9%, whereas 1.3% had indexed ICER (to 2020) of 100,000 US$.

**Figure 5:**
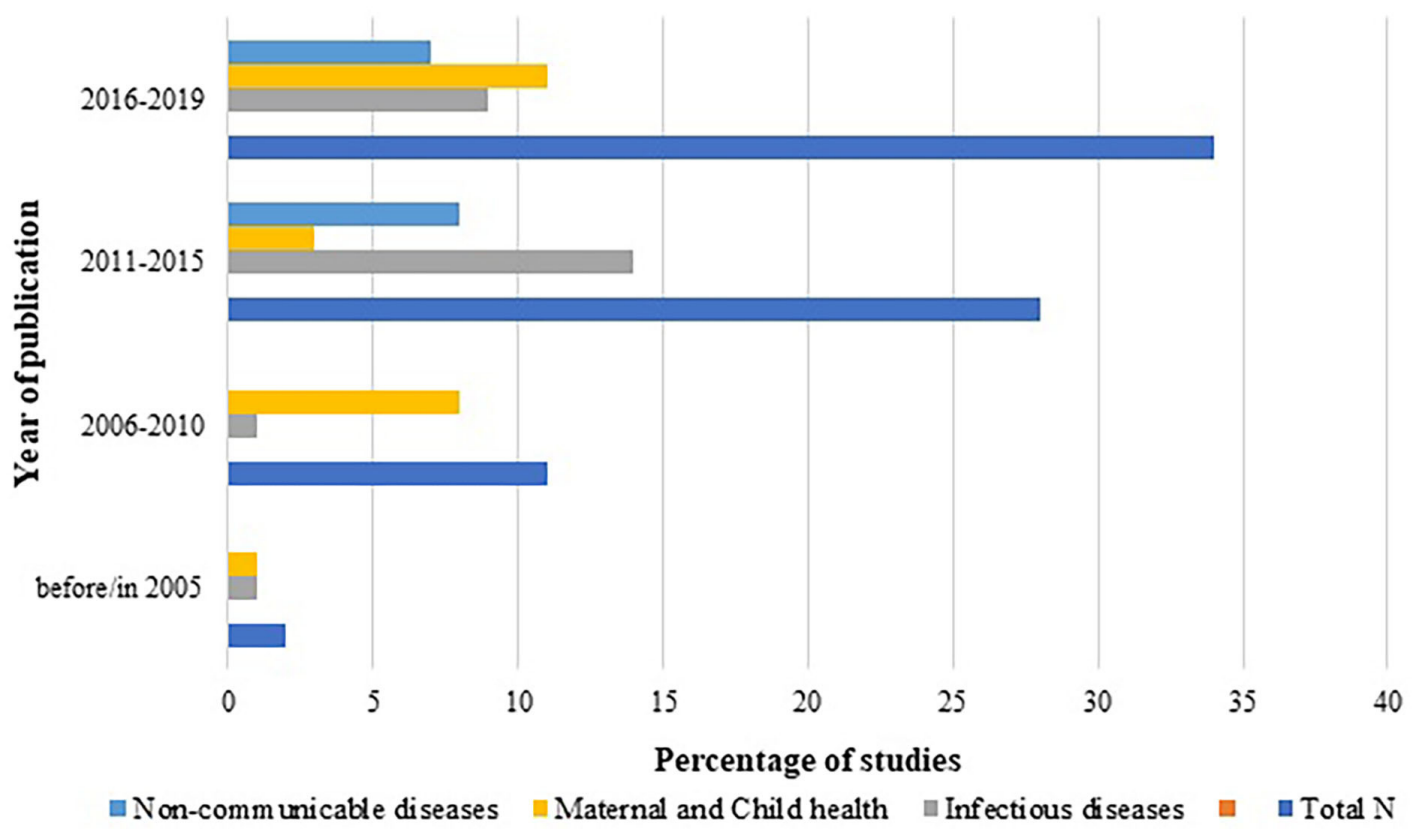
Number of HTA studies classified as per disease and year of publication.

Year-wise distribution of the studies according to the disease type was also assessed and plotted as Figure 5. It showed steep increase in HTA studies with time, with maximum HTA studies reported between 2015 to 2019. Although interventions for infectious diseases have shown consistently dominant trend among all the diseases across all the years, the last decade's innovations addressing non-communicable diseases have started picking up.

## Discussion

The prime challenge in development and calibration of league table is the heterogeneity among cost per QALY/DALY ratios reported by various studies. The purpose of this comprehensive review of published cost-effectiveness studies is to create a league table for India that may act as a reference document and assist in identify standardized methodologies and provide a landscape assessment of the of cost-effectiveness studies from India.

We compiled 79 studies reporting cost per QALY/DALY and observed that despite incremental cost-effectiveness ratio (ICER) varying widely, majority of the interventions were falling below the WHO recommended threshold of cost-effectiveness for India (per capita GDP 2019) (92). We retrieved and critically reviewed all the important methodological information from the 79 studies according to the reference case prepared by DHR for India (2, 19). The key exercises undertaken by the authors were the following: (1) The conversion of all the reported ICERs to corresponding value of 2020 US$. This served as an important base for “Head-to-Head Comparisons” of the interventions; (2) This league table provided entire spectrum of cost per QALY/DALY data for various disease groups that range from cost saving to 100,000 US$ per QALY/DALY and its distribution as per the perspective opted, along with the nature of care. This analysis yielded documentation of the group of interventions that are highly cost-effective; (3) The document also evaluated the quality of the finding and its potential use for informing policy decisions using surrogate markers, such as discounting, budget impact, and sensitivity analysis; (4) The league table also identified and mapped the interventions that are falling below the cost-effectiveness Indian threshold—per capita GDP (as per 2019 values) and categorized them further according to disease group and perspective of economic evaluation. The time trend analysis clearly depicted a steep increase in reporting of cost-effectiveness studies especially after the establishment of Health Technology Assessment in India (HTAIn)—an HTA body under DHR following the recommendation of 12th Plan Working Group on Health Research (93). This study may provide a detailed information and facilitate evidence-based decisions through parallel comparisons of the healthcare innovations. We aim to continuously upgrade the league tables with upcoming economic evaluations where the current report may act as a benchmark for future reference cases.

### Limitations

Although reporting of cost-utility ratios from India varies widely, the review restricted article inclusion using reporting criteria of most the popular forms—QALY and DALY—to provide better generalizability of the findings. In this process, some important studies, which expressed findings as cost per Life years saved (LYS) and other critical outcomes, remained untapped. Another important limitation of the league table presented here is to completely rely on the published cost—utility ratios, which may have some inherent bias and methodological challenges. There may be underreported clinical and modeling assumption that may significantly influence the outcome and interpretation. Moreover, some of the best pieces of research in gray literature are found in India that remain unpublished in reputed databases and, hence, we may have missed some of the important observations holding contextual values. In addition to this, we could not assess the quality of the studies using any of the recommended checklist due to considerable data gaps and had to adhere to the minimum criteria of quality assessment for compilation of current league table.

## Conclusion

With rapidly changing dynamics of healthcare investment, with establishment of India's own HTA body, increasing awareness about cost-effectiveness analysis of this snapshot of cost-utility studies will act as a valuable reference for healthcare planning and resource allocation. Limitations of existing health technology assessments or economic evaluation studies underscore methodological priorities for future health technology assessment studies. The disease specific league table will assist in mapping the disease burden with the investment needs and will prepare the country for combating economic burden associated with morbidity and mortality in futuristic manner.

## Data availability statement

The original contributions presented in the study are included in the article/Supplementary material, further inquiries can be directed to the corresponding author.

## Author contributions

KS and MS conceptualized and designed the study and developed search strategy. KS prepared first draft of the manuscript. KT, PK, and AP completed search, extracted data, and synthesize evidence. SS, DS, and KR guided evidence synthesis, resolve disputes in inclusion, exclusion of studies, reviewed, revised, and provided significant inputs in manuscript draft. All authors contributed to the article and approved the submitted version.

## Funding

This work was supported by the Health Technology Assessment in India, Department of Health Research, Ministry of Health and Family Welfare, India.

## Conflict of interest

The authors declare that the research was conducted in the absence of any commercial or financial relationships that could be construed as a potential conflict of interest.

## Publisher's note

All claims expressed in this article are solely those of the authors and do not necessarily represent those of their affiliated organizations, or those of the publisher, the editors and the reviewers. Any product that may be evaluated in this article, or claim that may be made by its manufacturer, is not guaranteed or endorsed by the publisher.
