# Supplementary material for: Comprehensive league table of cost-utility ratios: A systematic review of cost-effectiveness evidence for health policy decisions in India
Source: Front Public Health. 2022 Oct 13;10:831254. doi: 10.3389/fpubh.2022.831254 (PMC9606776; doi:10.3389/fpubh.2022.831254)
Supplement: Supplementary file 1 [file Table_1.docx]

**Supplementary Table 1: Cost-effectiveness league table of health interventions published from the year 2003 to 2019**

| **Sr. No** | **Author** | **Year** | **Disease/ Condition** | **Interventions** | **Comparator** | **Study perspective** | | **Cost-effective** | | | **Outcome measure** | | **ICER (Indexed to 2020)** | | | **Discount Rate** | | | **Budget Impact Analysis** | | **Type of Sensitivity Analysis** | | |
| --- | --- | --- | --- | --- | --- | --- | --- | --- | --- | --- | --- | --- | --- | --- | --- | --- | --- | --- | --- | --- | --- | --- | --- |
| **Infectious diseases** | | | | | | | | | | | | | | | | | | | | | | | |
| 1 | Ghoshal et al ^22^ | 2003 | Duodenal Ulcer hemorrhage | Maintenance therapy with proton pump inhibitors (PPIs) for the treatment of H. pylori infection | Empirical treatment for possible H. pylori infection  Maintenance  Eradication of H. pylori  Maintenance of PPI | Patient's Perspective | | Yes | | | QALY | | 215.15 | | | Not mentioned | | | No | | OWSA +  TWSA | | |
| 2 | Fung et al^23^ | 2007 | HIV/AIDS | HIV prevention programme with Female sex workers (FSW) | No intervention | Provider’s Perspective | | Yes | | | DALY | | 7.6 | | | 3% | | | No | | TWSA | | |
| 3 | Dandona et al^24^ | 2010 | HIV/AIDS | 128 HIV prevention programmes | No intervention | Health System Perspective | | Yes | | | DALY | | 488.59 | | | 3% | | | No | | Not conducted | | |
| 4 | Dowdy et al^25^ | 2011 | Tuberculosis | Sputum smear microscopy followed by mycobacterium growth indicator | Routine care | Health System Perspective | | Yes | | | DALY | | 249.73 | | | 3% | | | No | | OWSA | | |
| 5 | Goodchild et al^26^ | 2011 | Tuberculosis | Revised National Tuberculosis Control Programme | No intervention | Societal Perspective | | Yes | | | DALY | | 30.48 | | | 3% | | | No | | Unclear | | |
| 6 | Prinja et al^27^ | 2011 | HIV/AIDS | HIV prevention programme with Female sex workers (FSW) | Baseline scenario of mass media for the general population | Health System Perspective | | Yes | | | DALY | | 12.78 | | | 3% | | | No | | OWSA | | |
| **7** | Reid et al^28^ | 2012 | Blood borne diseases | Introduction of auto disable syringes | No intervention | Health System Perspective | | Yes | | | DALY | | 89.78 | | | Not mentioned | | | No | | Not conducted | | |
| 8 | Eaton et al^29^ | 2013 | HIV/AIDS | Anti-retroviral therapy (ART) to all HIV positive adults | Routine care | Health System Perspective | | Yes | | | DALY | | 322.91 | | | 3% | | | No | | Not conducted | | |
| 9 | Hoog et al^30^ | 2013 | Tuberculosis | Xpert MTB/RIF assay | Routine care | Health System Perspective | | Yes | | | DALY | | 748.27 | | | Not mentioned | | | No | | PSA | | |
| 10 | Vassall et al^31^ | 2014 | HIV/AIDS | HIV prevention programme with Female sex workers | Non-programme counterfactual scenario | Provider’s Perspective | | Yes | | | DALY | | 50.47 | | | 3% | | | No | | OWSA + PSA | | |
| 11 | Vassall et al^32^ | 2014 | HIV/AIDS | Community Mobilisation and Empowerment | Base case of providing just the core HIV prevention activities for FSWs HIV programme | Health system Perspective | | Yes | | | DALY | | 15.49 | | | 3% | | | No | | OWSA + PSA | | |
| 12 | Joshi et al^33^ | 2015 | HIV | Repeat HIV test | No repeat HIV test | Health system Perspective | | Yes | | | QALY | | 1055.03 | | | Not mentioned | | | No | | OWSA | | |
| 13 | Kelly et al^34^ | 2015 | Pulmonary Tuberculosis | LED Fluorescence Microscopy | ZN microscopy | Health system Perspective | | Yes | | | DALY | | 15.8 | | | Not mentioned | | | No | | OWSA | | |
| 14 | Little et al ^35^ | 2015 | Tuberculosis | Standard of care augmented by microbiological tests Xpert MTB/ RIF | Standard of Care without microbiological tests | Health system perspective | | Yes | | | DALY | | 77.50 | | | 3% | | | No | | OWSA + PSA | | |
| 15 | Maddali et al^36^ | 2015 | HIV | Early ART initiation | Routine care | Health system Perspective | | Yes | | | QALY | | 572.33 | | | 3% | | | No | | OWSA | | |
| 16 | Rosenthal et al^37^ | 2015 | Infection | Single-use prefilled flushing devices (SUFs) | Standard intravenous flushing | Health system Perspective | | Yes | | | QALY | | Cost saving | | | Not mentioned | | | No | | Not conducted | | |
| 17 | Suen et al^38^ | 2015 | Tuberculosis | GeneXpert and PPM delivered alone or in combination | Routine care | Societal Perspective | | Yes | | | QALY | | 1407.70 | | | 3% | | | Yes | | PSA | | |
| 18 | Kapoor et al^39^ | 2016 | HIV | Isoniazid preventive therapy (IPT) | No Isoniazid preventive therapy (IPT) | Health system Perspective | | Yes | | | DALY | | 216.79 | | | 3% | | | No | | PSA | | |
| 19 | Aggarwal et al^40^ | 2017 | Hepatitis C virus (HCV) infection | Generic directly-acting antivirals (DAA) | Branded directly-  acting antivirals  (DAA) | Payer's perspective | | Yes | | | QALY | | Cost saving | | | 3% | | | No | | OWSA+  PSA | | |
| 20 | John et al^41^ | 2017 | Multi Drug Resistant Tuberculosis | Decentralised care model | Centralized care model | Health care system's perspective | | Yes | | | QALY | | Cost saving | | | Not mentioned | | | No | | OWSA | | |
| 21 | Lu et al^42^ | 2017 | Multi Drug Resistant Tuberculosis | Addition of bedaquiline to current treatment | Routine care | Health system Perspective | | Yes | | | DALY | | Cost saving | | | 5% | | | No | | PSA | | |
| 22 | Goel et al^43^ | 2018 | HCV infection | Pan-genotypic - direct-acting antivirals (DAA) | Genotype-dependent DAA | Payer’s perspective | | Yes | | | QALY | | 246.38 | | | 3% | | | No | | PSA | | |
| 23 | Krishnamoorthy et al^44^ | 2019 | Pneumococcal disease | pneumococcal conjugate vaccine | Routine care | Health system Perspective | | Yes | | | DALY | | 2365.05 | | | 3% | | | No | | PSA | | |
| 24 | Chaillon et al^45^ | 2019 | Hepatitis C virus infection | HCV direct-acting antivirals (DAAs) | No intervention | Healthcare payer perspective | | Yes | | | QALY | | 1614.71 | | | 3% | | | Yes | | OWSA | | |
| 25 | Chugh et al^46^ | 2019 | Hepatitis C Virus Infection | Pan genotypic Sofosbuvir-Velpatasvir combination | Genotype dependent directly acting anti-viral drugs | Societal Perspective | | Yes | | | QALY | | Cost saving | | | 3% | | | Yes | | PSA +  USA | | |
| 26 | Sohn et al^47^ | 2019 | Tuberculosis | Decentralization of Xpert testing at PHC | Centralized Xpert testing at DTC | Health system Perspective | | Secondary | | | DALY | | Cost saving | | | 3% | | |  | | Not conducted | | |
| **Non-communicable disease** | | | | | | | | | | | | | | | | | | | | | | | |
| 27 | Schulman-Marcus et al^48^ | 2010 | Cardiovascular Disease | Cost-effectiveness of performing ECG | No intervention | Societal Perspective | | Yes | | | QALY | | 15.07 | | | 3% | | | No | | OWSA + PSA | | |
| 28 | Lohse et al^49^ | 2011 | Diabetes | Universal gestational diabetic screening | No intervention | Health system Perspective | | Yes | | | DALY | | 13.27 | | | 3% | | | No | | OWSA | | |
| 29 | Buttorff et al^50^ | 2012 | Mental Disorders | Task-shifting intervention for common mental disorders | Routine care | Societal Perspective | | Yes | | | QALY | | Cost saving | | | Not mentioned | | | No | | OWSA | | |
| 30 | Marseille et al^51^ | 2013 | Gestational Diabetes | Gestational diabetes screening/1000 women | Routine care | Societal Perspective | | Yes | | | DALY | | 1810 | | | 3% | | | No | | OWSA+ PSA | | |
| 31 | Rachapelle et al^52^ | 2013 | Diabetic Retinopathy | Telemedicine to Screen | Do Nothing | Societal Perspective | | Yes | | | QALY | | 1469.82 | | | 3% | | | No | | OWSA+ PSA | | |
| 32 | Home et al^53^ | 2015 | Insulin-naı¨ve people with type 2 diabetes | Insulin detemir with OGLD | Oral glucose-lowering drugs (OGLDs) | Health system Perspective | | Yes | | | DALY | | 763.47 | | | 3% | | | No | | Not conducted | | |
| 33 | Basu et al^54^ | 2015 | CVD | Expanded national insurance | Routine insurance coverage | Societal Perspective | | Yes | | | DALY | | 1221.33 | | | 3% | | | No | | Not conducted | | |
| 34 | Gupta et al^55^ | 2015 | Type 2 Diabetes | Biphasic insulin aspart 30 (BIAsp 30) | Biphasic human insulin 30 (BHI), insulin glargine (IGlar), or neutral protamine Hagedorn (NPH) insulin (all oral glucose-lowering drugs [OGLDs]) | Health system Perspective | | Yes | | | QALY | | 377.96 | | | Not mentioned | | | No | | Not conducted | | |
| 35 | Patel et al^56^ | 2015 | Chronic obstructive pulmonary disease | Ward-based NIV with concurrent standard treatment | Routine care | Societal Perspective | | Yes | | | QALY | | 65.87 | | | 5% | | | No | | TWSA | | |
| 36 | Basu et al^57^ | 2016 | Type 2 Diabetes | Benefit-based, tailored treatment (BTT) strategy | Treat-to-target (TTT) strategy | Societal Perspective | | Yes | | | DALY | | Cost saving | | | 3% | | | No | | Not conducted | | |
| 37 | Raykar et al^58^ | 2016 | Schizophrenia | Universal public finance | No intervention | Societal Perspective | | Yes | | | DALY | | 1.71 | | | Not mentioned | | | No | | Not conducted | | |
| 38 | Nadkarni et al^59^ | 2017 | Alcohol problems | Counselling for Alcohol Problems (CAP) with Enhanced usual care | Enhanced usual care | Health system and Societal Perspective | | Yes | | | QALY | | Cost saving | | | Not mentioned | | | No | | Not mentioned the type of sensitivity analysis | | |
| 39 | Prinja et al^60^ | 2017 | Cervical cancer | Human papillomavirus (HPV) vaccination | No intervention | Societal Perspective | | Yes | | | QALY | | 1.19 | | | 3% | | | Yes | | PSA | | |
| 40 | Prinja et al^61^ | 2017 | Multiple myeloma | Autologous stem cell transplantation (ASCT) along with high dose chemotherapy (HDC) | Conventional chemotherapy | Societal Perspective | | Yes | | | QALY | | 2565.96 | | | 5% | | | No | | OWSA | | |
| 41 | Praveen et al^62^ | 2018 | Hypertension | 1) Treating people with “hypertension” 2) treatment as per the new Indian NPCDCS guidelines;  3) treating people in the intermediate and high-risk categories (regardless of BP level); and  4) treating only those in the high-risk category (regardless of BP level) | Routine care | Health system Perspective | | Yes | | | DALY | | 622.22 | | | Not mentioned | | | No | | Not mentioned the type of sensitivity analysis | | |
| 42 | Lin et al^63^ | 2019 | CVD | Polypill | Routine care | Health system perspective | | Yes | | | DALY | | 377.07 | | | 3% | | | No | | OWSA + PSA | | |
| **Maternal and child health** | | | | | | | | | | | | | | | | | | | | | | | |
| 43 | Aggarwal et al^64^ | 2003 | Hepatitis B | Universal Hepatitis B immunization | No intervention | Societal Perspective | | | Yes | | QALY | | | 18.79 | | | 3% | | | No | | | PSA |
| 44 | Suraratdecha et al^65^ | 2006 | Japanese Encephalitis | Immunization with inactivated vaccine | Routine care | Societal Perspective | | | Yes | | DALY | | | 99.53 | | | 3% | | | No | | | OWSA |
| 45 | Goldie et al^66^ | 2008 | Human Papilloma Virus | HPV vaccination of young adolescent girls | Routine care | Health system Perspective | | | Yes | | DALY | | | 777.02 | | | 3% | | | No | | | OWSA |
| 46 | Cook et al^67^ | 2008 | Typhoid | Community based typhoid vaccination program | Baseline scenario | Health System and Societal Perspective | | | Yes | | DALY | | | 679.92 | | | 3% | | | Yes | | | OWSA+PSA |
| 47 | Esposito et al^68^ | 2008 | Gastroenteritis | National Rotavirus vaccination program | No intervention | Health System Perspective | | | Yes | | DALY | | | 26.4 | | | 3% | | | No | | | OWSA |
| 48 | Dabral et al^69^ | 2009 | Measles | Supplementary Immunization | No intervention | Provider’s Perspective | | | Yes | | DALY | | | 10.29 | | | 3% | | | No | | | OWSA +  TWSA + Three-way sensitivity analysis |
| 49 | Jeuland et al^70^ | 2009 | Cholera | Cholera vaccination: School based program (children in the age group of 1-14 years) | No intervention | Health System and Societal Perspective | | | Yes | | DALY | | | 3368.14 | | | 3% | | | No | | | OWSA |
| 50 | Rose et al^71^ | 2009 | Gastroenteritis | Mass Rotavirus vaccination for a birth cohort in India | No intervention | Health System and Societal Perspective | | | Yes | | DALY | | | 160.73 | | | 3% | | | Yes | | | OWSA+PSA |
| 51 | Sutherland et al^72^ | 2010 | PPH | Prophylactic misoprostol for the prevention of post-partum haemorrhage | Routine care | Health system Perspective | | | Yes | | DALY | | | 202.59 | | | 3% | | | No | | | Not conducted |
| 52 | Clark et al^73^ | 2013 | Pneumonia, meningitis, and  non pneumonia non-meningitis invasive diseases | Haemophilus influenzae Type b | No intervention | Societal Perspective | | | Yes | | DALY | | | 1045.57 | | | 3% | | | Yes | | | OWSA+PSA |
| 53 | Gupta et al^74^ | 2013 | Bacterial Meningitis, Bacterial Pneumonia | Introduction of Haemophilus influenzae type b vaccination in the universal immunization schedule | No intervention | Health System and Societal Perspective | | | Yes | | DALY | | | 328.98 | | | 3% | | | Yes | | | OWSA |
| 54 | Rheingans et al^75^ | 2014 | Rotavirus | Rotavirus vaccination | No intervention | Health system Perspective | | | Yes | | DALY | | | 129.49 | | | 3% | | | No | | | OWSA + PSA |
| 55 | Plessow et al^76^ | 2016 | Iron deficiency anemia | Price subsidies on fortified packaged infant cereals (F-PICs) | Routine care | Societal Perspective | | | Yes | | DALY | | | Cost saving | | | 3% | | | No | | | Not mentioned the type |
| 56 | Prinja et al^77^ | 2016 | Neonatal and Childhood Illnesses | IMNCI programme | Routine care | Societal  Perspective | | | Yes | | DALY | | | 37.26 | | | 3% | | | Yes | | | PSA |
| 57 | Fitzpatrick et al^78^ | 2016 | Rabies | One Health approach | No intervention | Societal Perspective | | | Yes | | DALY | | | 4035.75 | | | 3% | | | Yes | | | Not conducted |
| 58 | Antillón et al^79^ | 2017 | Typhoid - typhoid conjugate vaccines (TCVs) | Routine infant vaccination with or without catch-up campaigns | No intervention | Health system Perspective | | | Yes | | DALY | | | Cost saving | | | 3% | | | No | | | Not conducted |
| 59 | Sinha et al^80^ | 2017 | Maternal and new-born mortality | Participatory learning and action with women’s groups facilitated by Accredited Social Health Activists | Routine care | Health system Perspective | | | Yes | | DALY | | | 88.41 | | | 3% | | | Yes | | | OWSA |
| 60 | Zhang et al^81^ | 2017 | Childhood pneumonia | Childhood pneumonia management using the 2005 and 2013 WHO guidelines | Routine care | Provider's Perspective | | | Yes | | DALY | | | 134.07 | | | Not mentioned | | | No | | | Not conducted |
| 61 | Goudet et al^82^ | 2018 | Severe acute Malnutrition | Community- based management of severe acute malnutrition programmes | Routine care | Provider's Perspective | | | Yes | | DALY | | | 37.54 | | | 3% | | | No | | | Not conducted |
| 62 | Powell-Jackson et al^83^ | 2018 | Diphtheria, Pertussis, Tetanus (DPT) vaccine (DPT3) immunization | Intervention to provide health information on immunisation | No intervention | Provider’s Perspective | | | Yes | | DALY | | | 193.99 | | | 3% | | | No | | | Not conducted |
| 63 | Prinja et al^84^ | 2018 | Maternal and new-born mortality | mobile phone-based heath technology | Routine care | Societal Perspective | | | Yes | | DALY | | | 213.8 | | | 3% | | | Yes | | | PSA |
| 64 | Bettampadi et al^85^ | 2019 | Measles | Community health workers (ASHA) supported vaccination | Without ASHA vaccination programme | Societal Perspective | | | Yes | | DALY | | | 169 | | | 3% | | | No | | | OWSA +  Bivariate, multivariate sensitivity analysis |
| 65 | Kashi et al^86^ | 2019 | Anemia | Multiple micronutrient supplementation | Longstanding iron and folic acid supplementation (IFA) | Health system Perspective | | | Yes | | DALY | | | 34.1 | | | 3% | | | No | | | PSA |
| **Others** | | | | | | | | | | | | | | | | | | | | | | | |
| 66 | Frick et al^87^ | 2009 | Uncorrected refractive error | School based eye screening program per 1000 children | No intervention | Health System Perspective | Yes | | | DALY | | 6858.61 | | | Not mentioned | | | No | | | | OWSA+PSA | |
| 67 | Rob et al^88^ | 2009 | Hearing Impairment | Screening and delivery of hearing aids at tertiary level | Routine care | Health System and Societal Perspective | Yes | | | DALY | | 1068.87 | | | 3% | | | No | | | | OWSA | |
| 68 | Cecchini et al^89^ | 2010 | Obesity | Mass media campaigns for the control of obesity epidemic | Routine care | Societal Perspective | Yes | | | DALY | | 2979.22 | | | 3% | | | Yes | | | | PSA | |
| 69 | Chow et al^90^ | 2010 | Vitamin A deficiency | High dose vitamin A supplementation for treating vitamin A deficiency | Routine care | Health System Perspective | Yes | | | DALY | | 536.26 | | | 3% | | | Yes | | | | PSA | |
| 70 | Brown et al^91^ | 2012 | Smoking | School based smoking prevention program | No intervention | Societal Perspective | Yes | | | QALY | | 2337.88 | | | Not mentioned | | | No | | | | PSA | |
| 71 | Hackenberg et al^92^ | 2015 | Cleft lip and palate (CLP) defects | 1) comprehensive care centres (CCC) [a specialized health care centre for cleft lip and palate patients, which provides in-country continuity of care and offers nutrition, speech pathology, psychology, and dental services.]  2) medical missions [a short-term humanitarian operation, with an average span of one to two weeks, and largely independent of local medical infrastructure | No intervention | Payer's perspective | Yes | | | DALY | | 499.49 | | | 3% | | | No | | | | Not conducted | |
| 72 | Khan et al^93^ | 2015 | Visual function | Phacoemulsification (PE) | Manual small-incision cataract surgery (MSICS) | Societal Perspective | Yes | | | QALY | | 33.73 | | | Not mentioned | | | No | | | | Not conducted | |
| 73 | Megiddo et al^94^ | 2016 | Epilepsy | National epilepsy programs | No intervention | Societal Perspective | Yes | | | DALY | | 1930.65 | | | 3% | | | No | | | | Latin Hypercube Sampling sensitivity analysis | |
| 74 | Arora et al^95^ | 2017 | Spinal cord injury | Usual care with Telephone-based support | Routine care | Societal Perspective | Yes | | | QALY | | 2687.39 | | | Not mentioned | | | No | | | | Not conducted | |
| 75 | John et al^96^ | 2017 | Glaucoma | Community screening programme | No intervention | Health System Perspective | Yes | | | QALY | | 165.4 | | | 3% | | | No | | | | OWSA | |
| 76 | Herzel et al^97^ | 2018 | Snake bite | 1) Anti-venom and supportive care and  2) an anti-venom/  adjunct combination strategy with supportive care | Antivenom alone | Payer's Perspective | Yes | | | DALY | | Cost saving | | | 3% | | | No | | | | OWSA +  PSA | |
| 77 | John et al^98^ | 2018 | Thalassemia major | Hematopoietic Stem Cell Transplant (HSCT) | Lifelong regular transfusion chelation (TC) therapy | Societal Perspective | Yes | | | QALY | | 2625.68 | | | 3% | | | No | | | | Not conducted | |
| 78 | Mateti et al^99^ | 2018 | Haemodialysis | Pharmaceutical Care | Routine care | Societal Perspective | Not clear | | | QALY | | 3312.59 | | | Not mentioned | | | No | | | | Not conducted | |
| 79 | Emmett et al^100^ | 2019 | Sensorineural Hearing Loss | Cochlear implantation (CI)  with mainstream education and deaf education with sign language | No intervention | Health System Perspective | Yes | | | DALY | | 13968.33 | | | 3% | | | No | | | | Not conducted | |

PSA: probabilistic sensitivity analysis; OWSA: one-way sensitivity analysis; TWSA: two-way sensitivity analysis
